# CG-Based Stratification of 8-mers Highlights Functional Roles and Phylogenetic Divergence Markers

**DOI:** 10.3390/ijms26199477

**Published:** 2025-09-27

**Authors:** Guojun Liu, Hu Meng, Zhenhua Yang, Guoqing Liu, Yongqiang Xing, Ningkun Xiao

**Affiliations:** 1 School of Life Science and Technology, Inner Mongolia University of Science and Technology, Baotou 014020, China; 2 Inner Mongolia Key Laboratory of Life Health and Bioinformatics, Inner Mongolia University of Science and Technology, Baotou 014020, China; 3 School of Economics and Management, Inner Mongolia University of Science and Technology, Baotou 014020, China; 4 Department of Immunochemistry, Institution of Chemical Engineering, Ural Federal University, Yekaterinburg 620075, Russia; 5 Laboratory for Brain and Neurocognitive Development, Ural Federal University, Yekaterinburg 620075, Russia

**Keywords:** CG dinucleotide, k-mer distribution, sequence evolution, information-theoretic analysis

## Abstract

K-mer analysis is a powerful tool for understanding genome structure and evolution. A “k-mer” refers to a short DNA sequence made up of k nucleotides (where k is a specific integer), while an “m-mer” is a similar concept but with a shorter sequence length. The functional mechanisms of CG-containing k-mers, as well as their potential role in evolutionary processes, remain unclear. To explore this issue, we analyzed 8-mers in several species with varying genomic complexities and evolutionary divergences: *Homo sapiens*, *Saccharomyces cerevisiae*, *Bombyx mori*, *Ciona intestinalis*, *Danio rerio*, and *Caenorhabditis elegans*, which were grouped by CG dinucleotide content (0CG, 1CG, and 2CG). We examined the relative frequencies of shorter m-mers (with m = 3 and 4) within each CG-defined group, using information-theoretic, distance-based, and angular metrics. Our results show that 0CG motifs follow random patterns, while 1CG and 2CG motifs display significant deviations, likely due to functional constraints such as nucleosome-binding and CpG island association. The observed unimodal distribution of 8-mers arises from the convergence of the three CG-defined groups. Among them, the 2CG group shows the highest divergence in m-mer composition, followed by 1CG, reflecting varying degrees of selective pressure. Furthermore, species-specific differences in CG-classified 8-mer patterns could provide valuable insights into phylogenetic relationships. Through extensive comparison, we explore how CG content and sequence composition influence genomic organization and contribute to evolutionary divergence across different taxa. These findings deepen our understanding of short motif functions, genome organization, and sequence evolution.

## 1. Introduction

DNA sequences serve as the fundamental carriers of genetic information, involved in gene expression, regulation, protein modulation, and the selection of transcription and replication start sites [1,2,3,4,5]. A k-mer, defined as a nucleotide sequence of length k, offers a powerful way to decode the “language” of DNA. By sliding a window of k bases along a genome, researchers can capture the functional and structural characteristics embedded in local motifs [6,7,8,9]. Prior to large-scale sequencing, studies of k-mer distributions focused on probabilistic modeling [10]. For example, Bultrini et al. used the principal component analysis of pentanucleotide frequency distributions to reveal typical intronic vocabulary preferences in *Caenorhabditis elegans* and *Drosophila melanogaster* [11]. Although k-mers themselves are not inherently functional, their usage patterns reflect biochemical biases, mutational pressures, and evolutionary selection, offering valuable insights into genome function and evolution. Some k-mers have been linked to protein-binding affinities [12,13], nucleosome formation [14,15], and conserved distributions in untranslated regions (UTRs) [16].

Despite the increasing availability of genome data, research on k-mers with k ≥ 3 has largely concentrated on extremely rare or frequent k-mers [17,18,19,20,21]. Notably, the concept of “nullomers”—k-mers absent from genomes—has been explored across >1500 species, with potential applications proposed by Hampikian and Andersen [22]. The availability of complete genome sequences has enabled comparative analyses of k-mer distributions across species and genomic regions. Reinert et al. first formalized the concept of k-mer distribution, noting that most k-mers follow a normal distribution, while a minority conform to a Poisson distribution [23]. Chor et al. extended this work, analyzing 4 ≤ k ≤ 13 k-mers across more than 100 organisms and identifying both unimodal and multimodal patterns. For example, 8-mer distributions were typically unimodal in non-mammalian species such as zebrafish, while species like chickens and frogs showed multimodal patterns [24]. Such variation is observed not only across species but also within different functional regions of the same genome. For instance, 8-mer distributions tend to be unimodal in prokaryotes [25,26], but multimodal in humans and mice [27]. Similar trends have been reported for 6-mers, with multimodal distributions observed in mammals and chickens, but not in non-mammalian species or Arabidopsis thaliana [28]. In mammals, multimodal k-mer distributions correlate with specific G + C content ranges and CpG suppression [29,30], although exceptions exist—for example, Entamoeba shows CpG suppression without multimodality [31]. Stacey et al. found that CpG suppression contributes to multimodal 8-mer spectra in mammals, whereas bacterial genomes remain unimodal [27]. While numerous studies have explored the causes of unimodal and multimodal k-mer distributions, the true underlying mechanisms remain unclear.

Jia et al. attributed the human genome’s trimodal 8-mer distribution to three distinct groups of CG-content-defined k-mers: CG0 (no CG), CG1 (one CG), and CG2 (two or more CGs), a phenomenon termed the “Law of Independent Selection”. CG1 motifs are implicated in nucleosome positioning, while CG2 motifs form core units of CpG islands [32,33]. Rare k-mers also show strong associations with promoters and CpG islands and can be predicted using methods like rare-word clustering (RWC) [34]. In addition, periodic distributions of CG, GG, CC, and GC dinucleotides have been identified as nucleosome positioning signals [35,36]. Experimental studies confirm that CC, CG, GC, and GG dinucleotides are enriched near nucleosome centers, while AT-rich motifs are often nucleosome-repelling [13,37,38]. Nyamdavaa et al. developed a nucleosome feature index based on 15 preferred or rare trinucleotides from CG1-containing 8-mers, which aligned well with nucleosome occupancy profiles around human transcription start sites [39]. Therefore, studying the nucleotide frequencies of shorter m-mers (m = 3, m = 4) within 8-mers could provide valuable insights into the functional roles of motifs.

Yeast (*Saccharomyces cerevisiae*) is a single-celled organism, and its simpler regulatory landscape and conserved chromatin structure make it an ideal model for large-scale k-mer analysis [13]. In particular, its use is especially valuable for investigating the functional roles of k-mers containing CpG dinucleotides. *Caenorhabditis elegans* (*C. elegans*) is an invertebrate and multicellular organism. As a relatively simple model organism, it has a unique but functionally significant nervous system, which provides valuable biological insights when compared to more complex organisms. *Ciona intestinalis* (sea squirt), although a chordate and “distant cousin” of vertebrates, lacks the key structure of a vertebral column. This makes it a meaningful species for k-mer comparison studies, as it provides insights into genomic evolution without the complexity of a vertebral structure. The silkworm (*Bombyx mori*) is also an invertebrate but exhibits greater anatomical complexity than simpler invertebrates due to its complete metamorphosis. This increased complexity makes it relevant for comparative analysis, particularly in studying developmental processes that differ from those in other organisms. The zebrafish (*Danio rerio*) genome sequencing project is one of the three major goals of the Genome Reference Consortium (GRC) [40]. In recent years, zebrafish has become a popular model for vertebrate development and human genetic disease research. Compared to other vertebrates, zebrafish has over 6000 genetic mutations, which is one of its most notable advantages. This provides a unique opportunity for genetic research, offering additional directions and content for scientific investigation [41]. By comparing the distribution distances of 0CG, 1CG, and 2CG subset motifs in the 8-mer relative motif distribution data across the genomes of *Saccharomyces cerevisiae*, *Danio rerio*, *Caenorhabditis elegans*, *Bombyx mori*, and *Ciona intestinalis*, we may gain valuable insights into the evolutionary divergence and functional roles of CG-containing motifs in different species.

In this study, we first analyzed the trimodal 8-mer distribution of human chromosome 1 and the unimodal distribution across the *Saccharomyces cerevisiae* genome, exploring the potential functions of CG0, CG1, and CG2-defined sequence groups. To uncover the sequence determinants underlying the unimodal 8-mer distribution observed in *Saccharomyces cerevisiae*, we applied New Symmetric Relative Entropy (NSRE), distance difference, and angle deviation metrics to assess the usage of 3-mers and 4-mers within each CG-defined 8-mer group. Furthermore, we calculated the peak distances between CG0, CG1, and CG2 subsets across *Saccharomyces cerevisiae*, *Bombyx mori*, *Ciona intestinalis*, *Danio rerio*, and *Caenorhabditis elegans*, in order to test the association between CG-based classification and evolutionary divergence among species with unimodal distribution patterns. Our analysis reveals differences in CG-content distribution across these species, providing insights into their unique genomic structures and functional constraints. This study underscores the importance of species diversity in understanding the evolutionary significance of k-mers and their potential role in genome organization.

## 2. Results

### 2.1. 8-mer Distribution in Human Chromosome 1

The genome assembly versions, total sequence lengths, and chromosome counts for these species are summarized in Table 1. Chor et al. reported that the trimodal distribution of k-mer spectra (for k = 4 to 13) becomes increasingly stable when k ≥ 6. They found that 6-mer spectra exhibit a unimodal pattern in bacteria, non-mammalian species, and Arabidopsis thaliana, but are multimodal in the genomes of human, mouse, and chicken [24]. In our study, we selected k = 8. This choice is based on Benny Chor’s recommendation to use the smallest k value satisfying the equation k = 0.7 log_4_ L, where L is the length of the DNA sequence. For the shortest protein-coding sequences in the human genome, the resulting minimum k value is approximately 8.9. To reduce local noise and visualize the overall trend of 8-mer frequency distributions, we applied a moving average smoothing with a window size of k = 10 using the rollmean function from the zoo R package (version 1.8.12).

**Figure 1:**
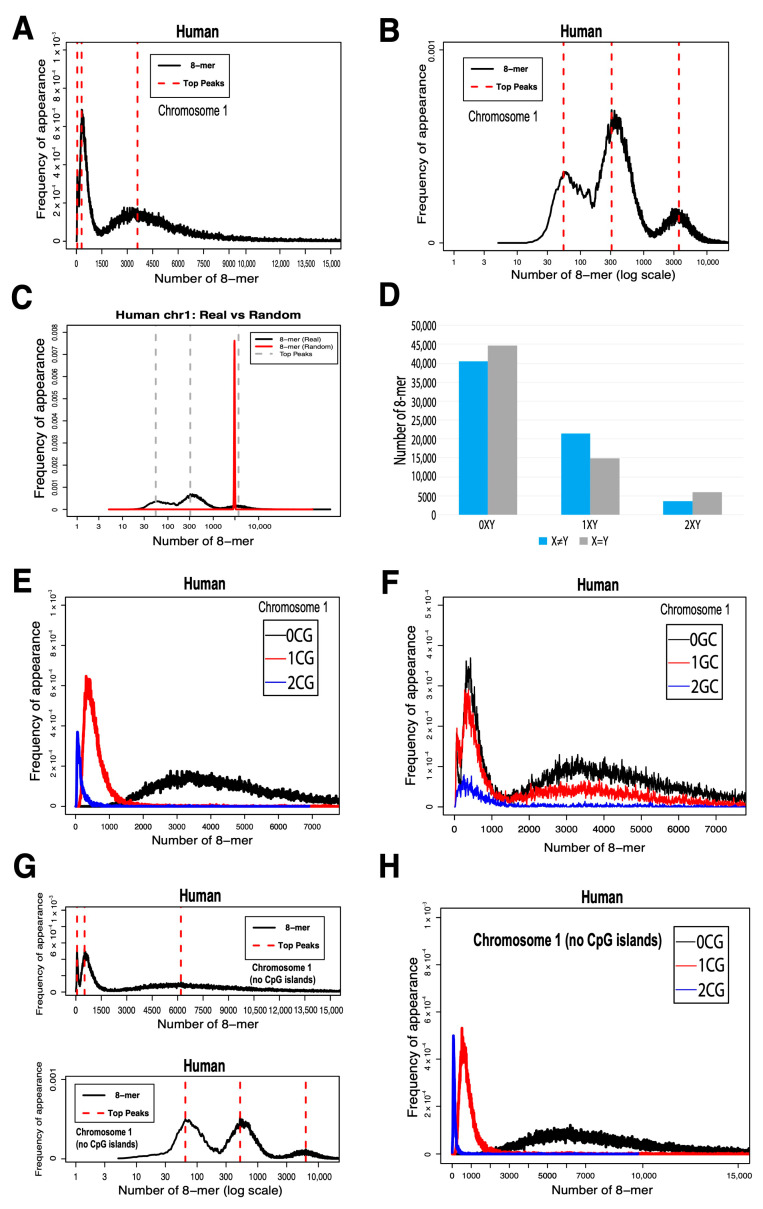
Distribution patterns of 8-mer frequencies in human chromosome 1 and analysis based on dinucleotide composition. (**A**) The distribution of 8-mers in human chromosome 1, with the x axis representing the number of k-mer appearances and the y axis indicating frequency of appearance (FA). (**B**) The same distribution shown with a log-transformed x axis. (**C**) A comparison with a random sequence of matched length and CG content shows that Peak3 corresponds to random 8-mer usage. (**D**) The number of 8-mers in the 0XY, 1XY, and 2XY subsets. (**E**) Distributions of 8-mers containing different numbers of CG dinucleotides. (**F**) Distributions of 8-mers containing different numbers of GC dinucleotides. (**G**) The distribution of 8-mers in chromosome 1 after removing CpG island sequences. (**H**) The same distribution as (**G**), shown with a log-transformed x axis.

As shown in Figure 1A, the *x* axis represents the 8-mer appearance from smallest to largest, and the y axis represents the relative motif count of the 8-mers, which is the frequency of appearance (FA) calculated using Formula (1). As a result, we obtained the FA distribution of 8-mers in the human chromosome 1 sequences, which showed a distinct trimodal distribution pattern (Figure 1A). For clearer visualization, the x axis of the distribution plot was transformed into a logarithmic scale (Figure 1B). The three peaks, labeled as Peak1, Peak2, and Peak3 from left to right, each contained a significant number of instances, with their central frequency ratios approximately being 50:307:3602. To investigate further, we generated a random sequence with the same length and CG content as the chromosome 1 sequence and plotted the relative frequency distributions of 8-mers for both the chromosome 1 and random sequences. Upon comparison, we observed that the random sequence (red) corresponded to Peak3, whereas the centers of Peak1 and Peak2 were significantly separated from that of the random sequence (Figure 1C). The complete set of 8-mers (which contains 4^8^ = 65,536 unique sequences) was divided into three subsets based on the number of 16 types of dinucleotides they contain—specifically, 0XY, 1XY, and 2XY—where X and Y represent the four nucleotide bases: A, C, G, and T. The 0XY subset includes 8-mer motifs that do not contain a specific dinucleotide; the 1XY subset includes motifs that contain exactly one occurrence of that dinucleotide; and the 2XY subset includes motifs with two or more occurrences (Figure 1D). The number of 8-mers in the three XY subsets varies depending on whether X and Y are the same or different.

After dividing the total 8-mers into these three subsets, we obtained the frequency distribution for each. We found that only the 8-mer distribution categorized by the CG dinucleotide formed independent peaks that were mutually exclusive and well-separated. We refer to this pattern as the independent distribution, as shown in Figure 1E. In contrast, for the other 15 dinucleotide categories, the 8-mer distributions formed overlapping peaks, which we defined as the non-independent distribution. For these non-independent distributions, only the motif distribution categorized by GC is presented, as the other 14 categories exhibited similar patterns (Figure 1F). We applied criteria based on GC content and the observed/expected ratio of CpG dinucleotides to identify and exclude CpG island sequences. First, the GC content of each sequence on human chromosome 1 was calculated, and sequences with a GC content below 50% were removed, as they do not exhibit typical CpG island features. Second, the observed/expected ratio of CpG dinucleotides was computed for each sequence. Sequences with a ratio below 0.6 were excluded, as their CpG density was not significantly higher than random expectation. Finally, additional filtering was performed based on sequence length and genomic location. We defined a sliding window approach with a window size of 1000 bp and a step size of 500 bp, and excluded short sequences located within gene bodies. The resulting 8-mer distribution after CpG island removal is shown in Figure 1G.

By comparing Figure 1H with Figure 1E, we observed a marked change in the distribution of 8-mers containing two CpG dinucleotides (2CG) after CpG island removal. These findings suggest that the usage of 8-mers with zero CpG dinucleotides (0CG) may reflect random evolutionary processes, whereas 2CG 8-mers represent core motifs contributing to CpG island formation.

### 2.2. Distribution of 8-mer Frequency of Appearance in Yeast

**Figure 2:**
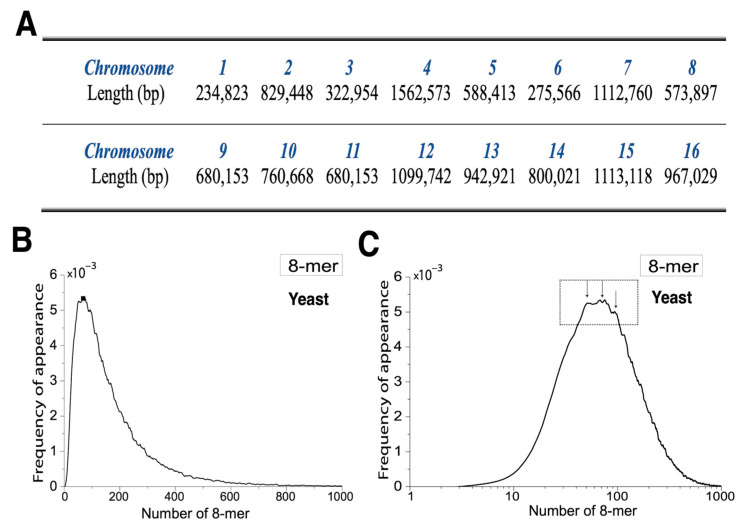
The 8-mer distribution in yeast genome sequences. (**A**) The sequence lengths of the sixteen chromosomes in yeast. (**B**) The unimodal distribution of 8-mers in the yeast genome sequence, with the x axis representing the number of k-mer appearances. (**C**) The unimodal distribution of 8-mers in the yeast genome sequence, with the x axis representing the logarithmic scale of the number of k-mer appearances.

The lengths of the 16 yeast chromosomes are shown in Figure 2A. To better illustrate the distribution pattern, we set the bin size to 1 and defined the x axis range from 1 to 1000. After multiple rounds of smoothing, the final distribution was obtained. The FA distribution of yeast 8-mers approximates a Poisson distribution and is positively skewed, indicating that the influence of low-frequency 8-mers (on the left side) is significantly greater than that of high-frequency 8-mers (on the right), resulting in an asymmetric pattern (Figure 2B). To enhance peak visibility, we applied a base-10 logarithmic transformation to the x axis. Upon closer inspection, the peak regions displayed a distinct sawtooth-like pattern (Figure 2C). Due to positive skewness, the slope on the left side is much steeper than that on the right, reflecting a much higher number of 8-mers with a lower occurrence than those with high frequency. To further investigate this trend, we extracted the least and most frequent 8-mers from the distribution (Table S1). The results revealed that 8-mer appearance counts were discontinuous: the least frequent 8-mers were predominantly rich in C and G bases, whereas the most frequent ones were primarily composed of A and T bases.

### 2.3. The 8-mer Distribution of Yeast Based on the XY Dinucleotide Classification

A total of 65,536 8-mers were first grouped into three subsets—0XY, 1XY, and 2XY—based on the number of occurrences of the 16 possible dinucleotides they contained. Using Formula (1), we calculated the FA distributions for each of the 16 subsets. The peak value of the FA distribution for the 0XY subset was denoted as FA0(p), with the corresponding appearance value denoted as N0(p). Similarly, FA1(p) and N1(p) represented the peak FA and the corresponding appearance for the 1XY subset, and FA2(p) and N2(p) for the 2XY subset. Overall, the 16 distribution plots could be broadly classified into three distinct categories based on their distributional patterns.

**Figure 3:**
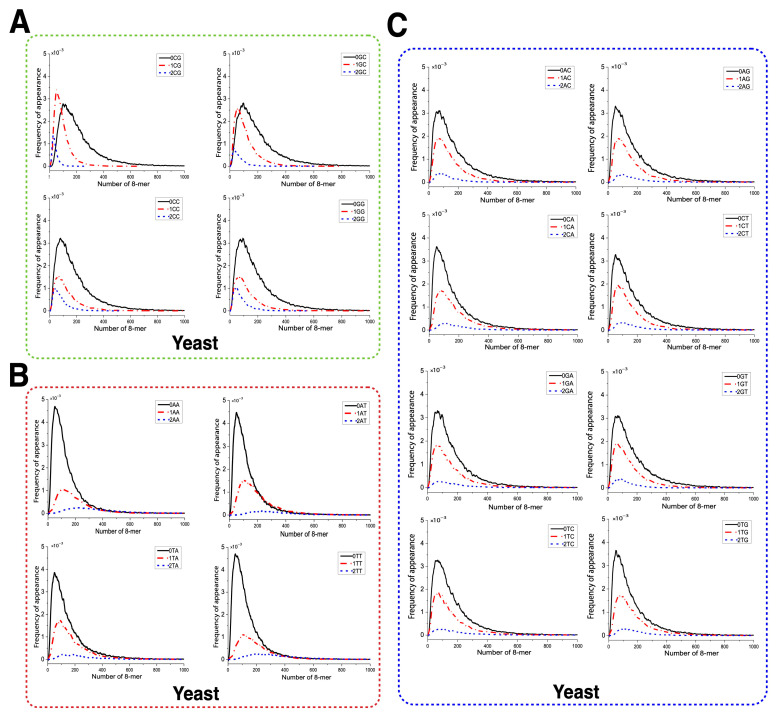
Distribution of 8-mers containing different counts (0, 1, 2) of the 16 dinucleotide types. (**A**) Distribution of 8-mers containing 0, 1, or 2 instances of CG, GC, CC, and GG dinucleotides. (**B**) Distribution of 8-mers containing 0, 1, or 2 instances of AA, AT, TA, and TT dinucleotides. (**C**) Distribution of 8-mers containing 0, 1, or 2 instances of each of the following dinucleotides: AC, AG, CA, CT, GA, GT, TC, and TG.

Figure 3A presents the 8-mer distributions based on the CG, GC, CC, and GG dinucleotide classifications. In all four cases, we observed that N2(p) < N1(p) < N0(p), indicating that the 2XY and 1XY subsets are left-shifted relative to the 0XY subset. For the GC, GG, and CC classifications, the distributions followed the pattern FA2(p) < FA1(p) < FA0(p), whereas in the CG classification, the pattern was FA2(p) < FA0(p) < FA1(p). This suggests that only the 1CG distribution exhibits a higher peak FA than both the 0CG and 2CG distributions, which is consistent with the pattern observed in human 8-mer distributions. Additionally, the 2CG distribution showed a higher peak FA than other 2XY distributions.

Figure 3B shows the 8-mer distributions for the AA, AT, TA, and TT dinucleotide classifications. In these cases, N0(p) < N1(p) < N2(p), indicating that the 1XY and 2XY subsets are shifted to the right relative to the 0XY subset. The distributions also followed the pattern FA2(p) < FA1(p) < FA0(p), with markedly lower peak FA values for the 1XY and 2XY subsets. Figure 3C presents the distributions for the AC, AG, CA, CT, GA, GT, TC, and TG classifications. Here, N0(p) ≈ N1(p) ≈ N2(p), suggesting minimal positional shift among subsets. However, the peak FA values still followed the trend FA2(p) < FA1(p) < FA0(p), with 1XY and 2XY subsets showing substantially lower peaks than 0XY.

### 2.4. Comparison of m-mer Usage in 8-mers Classified by 0XY, 1XY, 2XY

**Figure 4:**
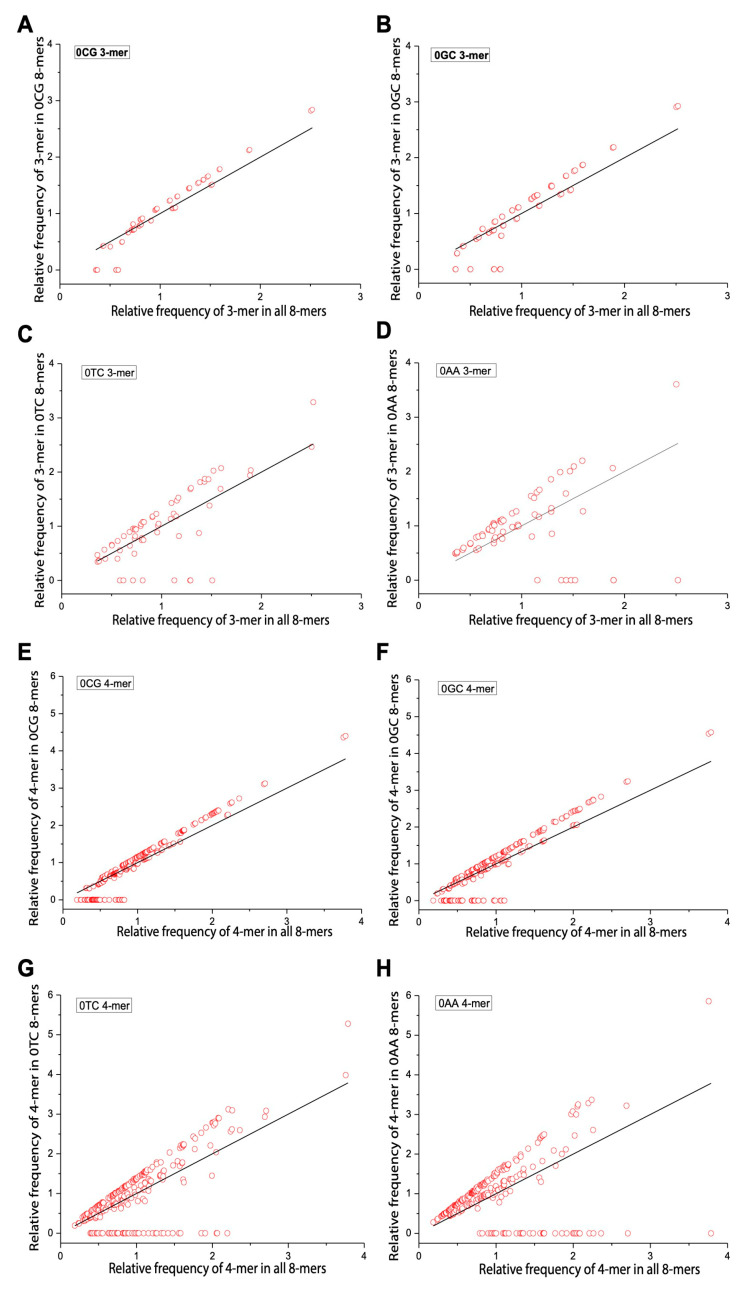
Usage divergence of 3-mers and 4-mers between 0CG, 0GC, 0TC, 0AA subsets and the overall 8-mer. (**A**) The usage divergence of 3-mers between the 0CG subset and the overall 8-mer. (**B**) The usage divergence of 3-mers between the 0GC subset and the overall 8-mer. (**C**) The usage divergence of 3-mers between the 0TC subset and the overall 8-mer. (**D**) The usage divergence of 3-mers between the 0AA subset and the overall 8-mer. (**E**) The usage divergence of 4-mers between the 0CG subset and the overall 8-mer. (**F**) The usage divergence of 4-mers between 0GC subset and the overall 8-mer. (**G**) The usage divergence of 4-mers between 0TC subset and the overall 8-mer. (**H**) The usage divergence of 4-mers between the 0AA subset and the overall 8-mer.

**Figure 5:**
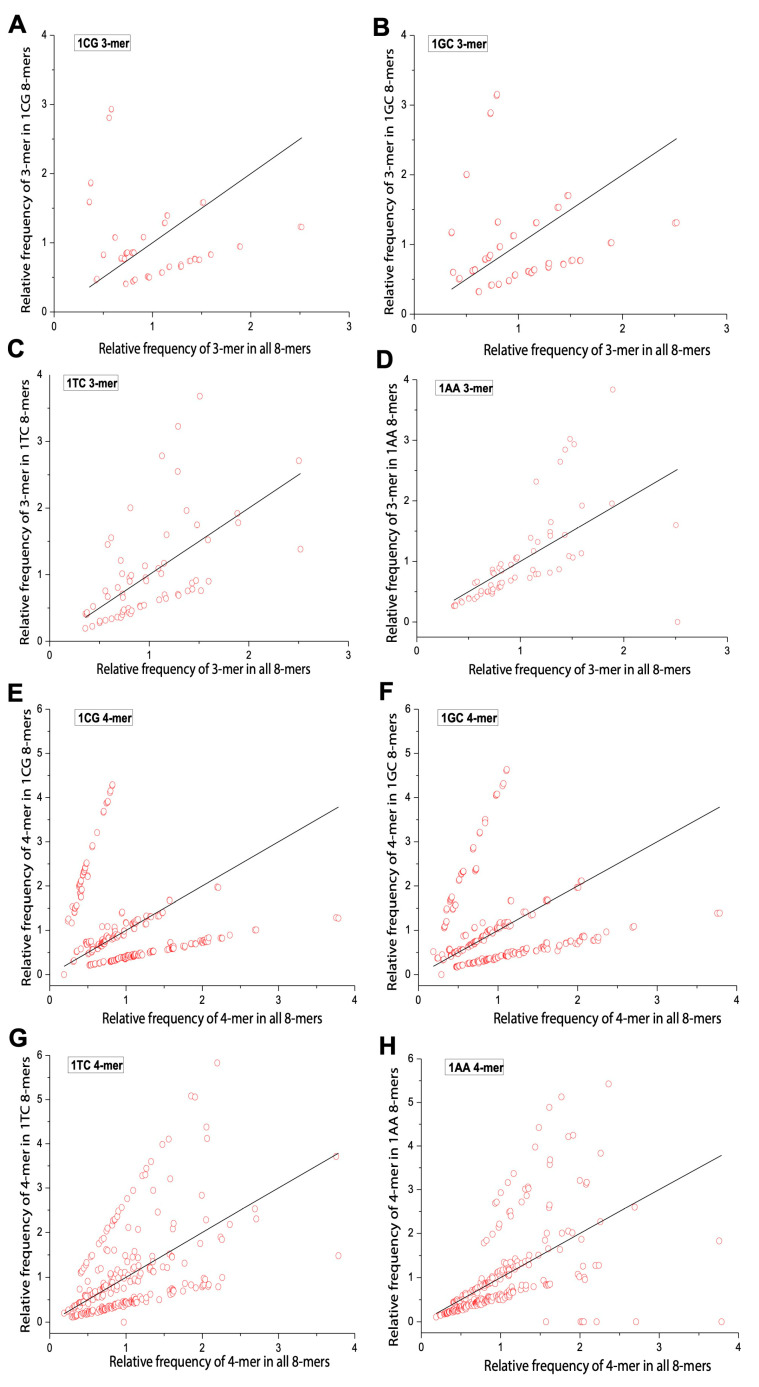
Usage divergence of 3-mer and 4-mer between the 1CG, 1GC, 1TC, and 1AA subsets and overall 8-mer. (**A**) The usage divergence of 3-mers between the 1CG subset and overall 8-mer. (**B**) The usage divergence of 3-mers between the 1GC subset and overall 8-mer. (**C**) The usage divergence of 3-mers between the 1TC subset and overall 8-mer. (**D**) The usage divergence of 3-mers between the 1AA subset and overall 8-mer. (**E**) The usage divergence of 4-mers between the 1CG subset and overall 8-mer. (**F**) The usage divergence of 4-mers between the 1GC subset and overall 8-mer. (**G**) The usage divergence of 4-mers between the 1TC subset and overall 8-mer. (**H**) The usage divergence of 4-mers between the 1AA subset and overall 8-mer.

**Figure 6:**
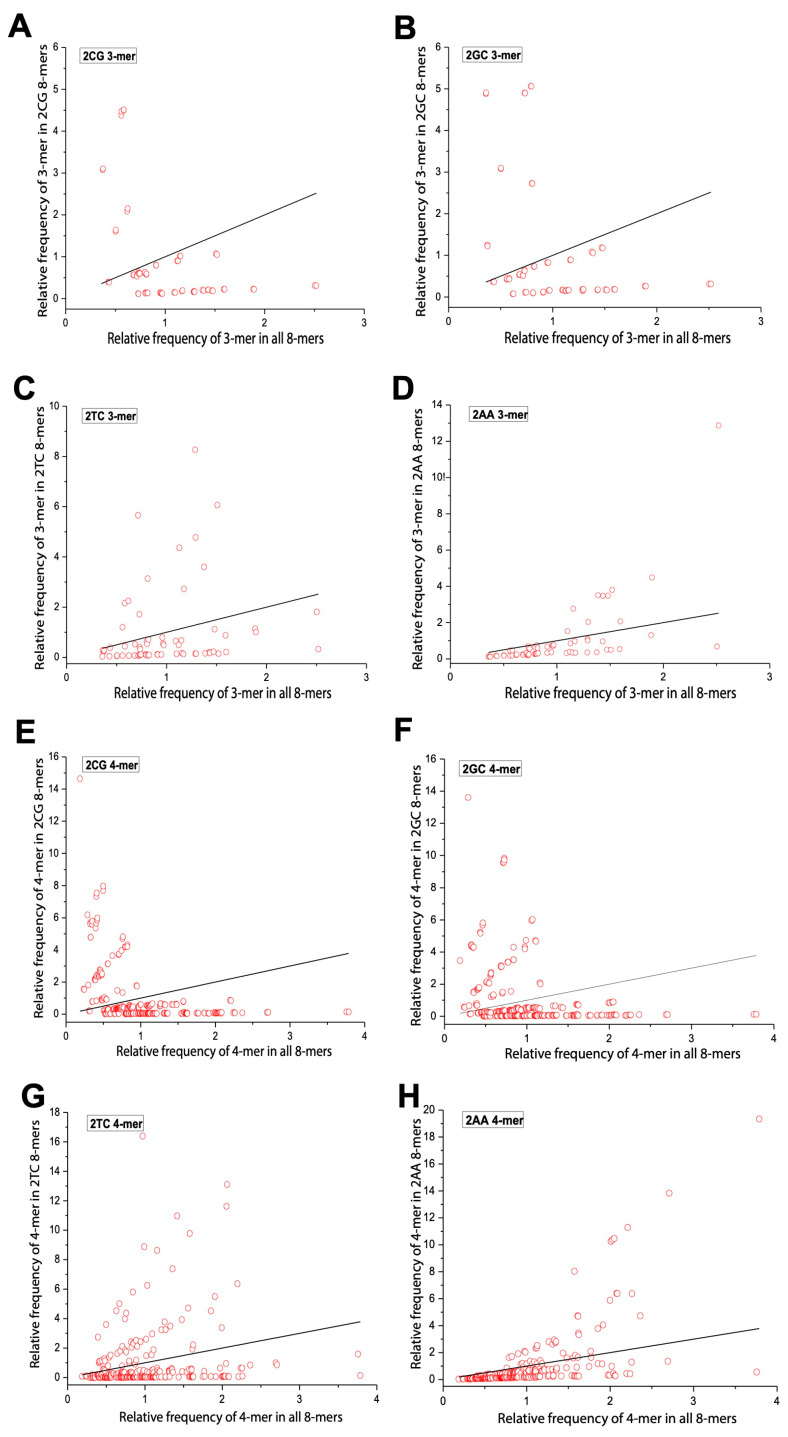
Usage divergence of 3-mer and 4-mer between the 2CG, 2GC, 2TC, and 2AA subsets and overall 8-mer. (**A**) The usage divergence of 3-mers between the 2CG subset and overall 8-mer. (**B**) The usage divergence of 3-mers between the 2GC subset and overall 8-mer. (**C**) The usage divergence of 3-mers between the 2TC subset and overall 8-mer. (**D**) The usage divergence of 3-mers between the 2AA subset and overall 8-mer. (**E**) The usage divergence of 4-mers between the 2CG subset and overall 8-mer. (**F**) The usage divergence of 4-mers between the 2GC subset and overall 8-mer. (**G**) The usage divergence of 4-mers between the 2TC subset and overall 8-mer. (**H**) The usage divergence of 4-mers between the 2AA subset and overall 8-mer.

We applied Formula (2) to calculate the relative frequencies of 3-mers and 4-mers in the overall 8-mer set. Table 2 shows all 64 3-mers, while Table 3 presents the top 15 (optimal) and bottom 15 (rare) 4-mers. Low-frequency motifs were enriched in C/G, and high-frequency ones were enriched in A/T. We then compared the relative frequencies of m-mers (m = 3, 4) in yeast 0XY, 1XY, and 2XY subsets against the overall set. To illustrate m-mer usage separation, we selected CG and GC (small separation), AA (large separation), and TC (moderate separation) subsets. The x axis represents m-mer frequencies in the overall set, and the y axis shows their frequencies in each subset. Red circles denote m-mers, and deviation from the diagonal indicates separation. In 0XY subsets, most m-mers formed two clusters—one near the diagonal and one oblique. 0CG/0GC showed the least separation, and 0AA showed the most (Figure 4). In 1XY subsets, m-mers formed three clusters, with 1CG/1GC showing the largest separation and 1AA showing the smallest (Figure 5). In 2XY subsets, most m-mers clustered in the lower-left; 2CG/2GC exhibited the strongest deviations, and 2AA exhibited the weakest (Figure 6). Overall, m-mer usage separation increased from 0XY to 2XY, suggesting stronger directed evolution in 2XY.

### 2.5. Differences in m-mer Usage Based on NSRE

**Figure 7:**
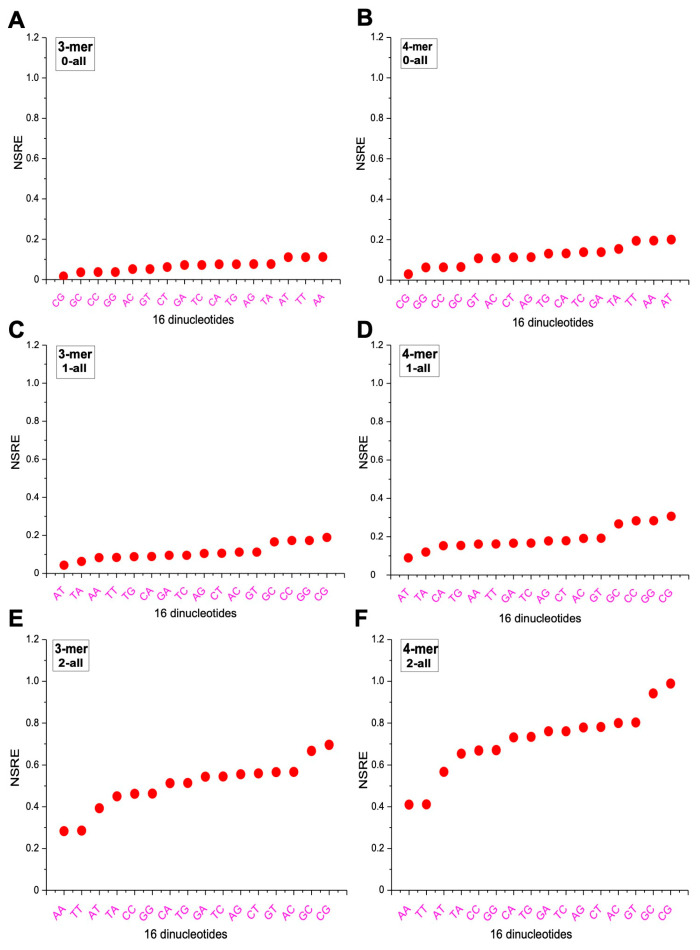
Analysis of the usage divergence of 3-mers and 4-mers between the 0XY, 1XY, and 2XY subsets and the overall 8-mer based on NSRE. (**A**) Analysis of the usage divergence of 3-mers between the 0XY subset and the overall 8-mer based on NSRE. (**B**) Analysis of the usage divergence of 4-mers between the 0XY subset and the overall 8-mer based on NSRE. (**C**) Analysis of the usage divergence of 3-mers between the 1XY subset and the overall 8-mer based on NSRE. (**D**) Analysis of the usage divergence of 4-mers between the 1XY subset and the overall 8-mer based on NSRE. (**E**) Analysis of the usage divergence of 3-mers between the 2XY subset and the overall 8-mer based on NSRE. (**F**) Analysis of the usage divergence of 4-mers between the 2XY subset and the overall 8-mer based on NSRE.

We adopted a previously defined metric, NSRE(P‖Q), to quantify the separation distance of 3-mers and 4-mers along the same angular direction. For m-mers located at the same angle, NSRE(P‖Q) increases with their distance from the origin. For example, if the frequency of AAA is 2 in the overall 8-mer set and 3 in a subset, while CCC has frequencies of 1 and 1.5, respectively, then the NSRE(P‖Q) value of AAA is twice that of CCC, reflecting a greater deviation. We calculated the NSRE(P‖Q) values for all 3-mers and 4-mers in the 0XY, 1XY, and 2XY subsets relative to the overall 8-mer set, and grouped the results into six categories (Figure 7). In the plots, the x axis represents the 16 dinucleotide categories, and the y axis shows the corresponding NSRE(P‖Q) values. In the 0XY subset, m-mers associated with the AA, TT, and AT categories had the highest NSRE(P‖Q) values, indicating the greatest deviation from the overall distribution, while those in the CG, GC, CC, and GG categories exhibited the lowest (Figure 7A,B). In the 1XY subset, the highest NSRE(P‖Q) values were observed for m-mers in the CG, GC, CC, and GG categories, while the AT and TA categories showed the lowest deviation (Figure 7C,D). In the 2XY subset, m-mers in the CG and GC categories displayed the largest deviations, whereas those in the AA and TT categories had the smallest (Figure 7E,F). Overall, the differences in NSRE(P‖Q) values were most pronounced in the 2XY subset, moderate in the 1XY subset, and least evident in the 0XY subset.

### 2.6. Characterization of m-mer Usage Bias Based on Distance Differences

**Figure 8:**
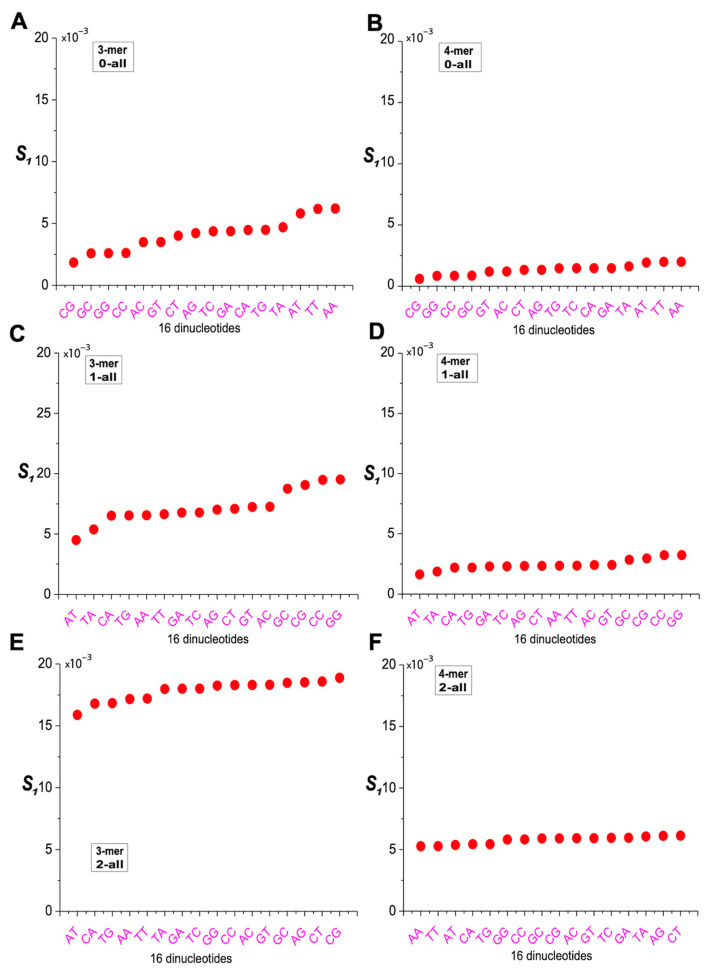
Analysis of the usage divergence of 3-mers and 4-mers between the 0XY, 1XY, and 2XY subsets and the overall 8-mer based on S_1_. (**A**) Analysis of the usage divergence of 3-mers between the 0XY subset and the overall 8-mer based on S_1_. (**B**) Analysis of the usage divergence of 4-mers between the 0XY subset and the overall 8-mer based on S_1_. (**C**) Analysis of the usage divergence of 3-mers between the 1XY subset and the overall 8-mer based on S_1_. (**D**) Analysis of the usage divergence of 4-mers between the 1XY subset and the overall 8-mer based on S_1_. (**E**) Analysis of the usage divergence of 3-mers between the 2XY subset and the overall 8-mer based on S_1_. (**F**) Analysis of the usage divergence of 4-mers between the 2XY subset and the overall 8-mer based on S_1_.

Compared to the overall 8-mer sequence, in the 0XY subset, m-mers from the AA, TT, and AT categories showed significantly higher S_1_ values, while those from the CG, GC, CC, and GG categories showed significantly lower values, indicating that 0AA-, 0TT-, and 0AT-containing 8-mers deviated the most and 0CG-, 0GC-, 0CC-, and 0GG-containing 8-mers deviated the least (Figure 8A,B). In the 1XY subset, m-mers from the CG, GC, CC, and GG categories had the highest S_1_ values, and those from the AT/TA categories had the lowest, suggesting that 1CG-, 1GC-, 1CC-, and 1GG-containing 8-mers deviated the most, while 1AT- and 1TA-containing 8-mers deviated the least (Figure 8C,D). In the 2XY subset, the S_1_ values of 3-mers and 4-mers formed an oblique line, indicating smaller differences across the 16 dinucleotide categories, with significant variation among subsets but no consistent pattern (Figure 8E,F). Overall, the 0XY subset exhibited the lowest S_1_ values, indicating the least deviation from the overall 8-mer sequence, whereas the 2XY subset showed the highest S_1_ values, reflecting the greatest deviation. The 1XY subset displayed intermediate S_1_ values between the two.

### 2.7. Characterization of m-mer Usage Bias Based on Angular Deviation

**Figure 9:**
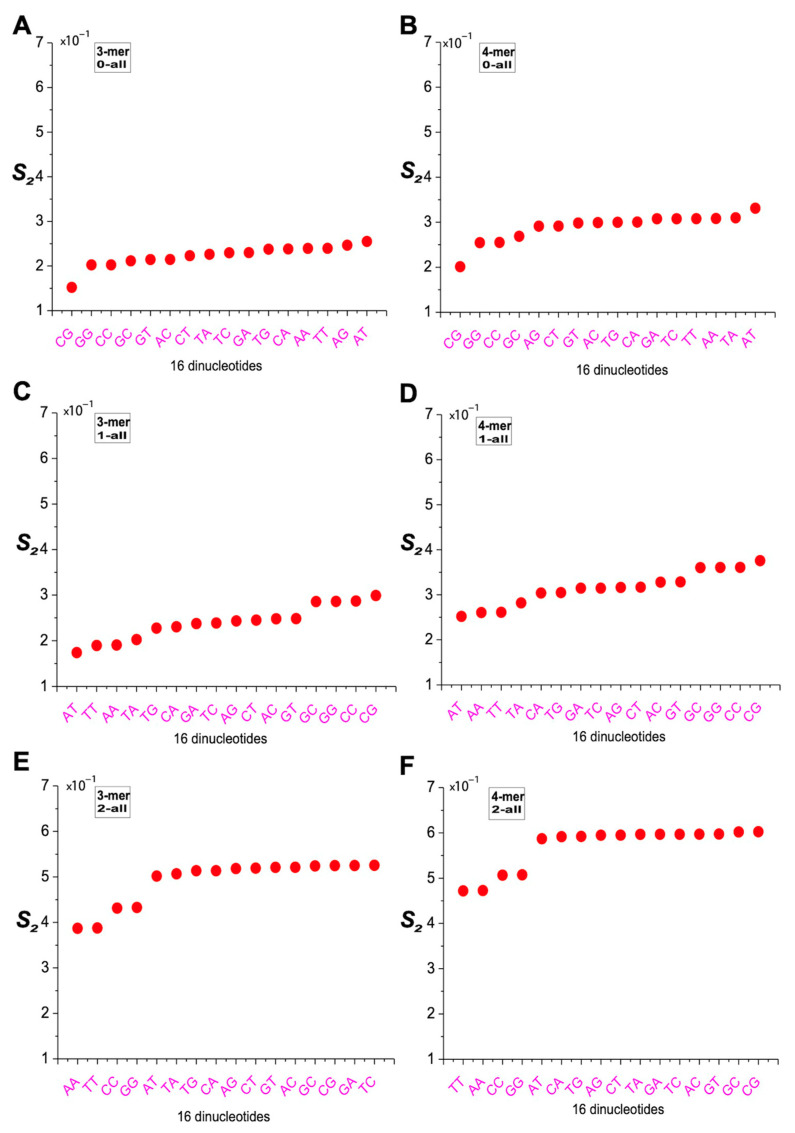
Analysis of the usage divergence of 3-mers and 4-mers between the 0XY, 1XY, and 2XY subsets and the overall 8-mer based on S_2_. (**A**) Analysis of the usage divergence of 3-mers between the 0XY subset and the overall 8-mer based on S_2_. (**B**) Analysis of the usage divergence of 4-mers between the 0XY subset and the overall 8-mer based on S_2_. (**C**) Analysis of the usage divergence of 3-mers between the 1XY subset and the overall 8-mer based on S_2_. (**D**) Analysis of the usage divergence of 4-mers between the 1XY subset and the overall 8-mer based on S_2_. (**E**) Analysis of the usage divergence of 3-mers between the 2XY subset and the overall 8-mer based on S_2_. (**F**) Analysis of the usage divergence of 4-mers between the 2XY subset and the overall 8-mer based on S_2_.

An analysis of m-mer usage differences based on angular deviations revealed that, in the 0XY subset, the S_2_ values for the AT category were significantly higher, while those for the CG, GC, CC, and GG categories were lower. This indicates that 8-mers containing 0AT deviated the most, whereas those containing 0CG, 0GC, 0CC, and 0GG deviated the least from the overall 8-mer sequence (Figure 9A,B). In the 1XY subset, the CG, GC, CC, and GG categories exhibited the highest S_2_ values, while the AT, TA, AA, and TT categories showed the lowest. These results suggest that 8-mers containing 1CG, 1GC, 1CC, and 1GG deviated the most, while those containing 1AT, 1TA, 1AA, and 1TT deviated the least (Figure 9C,D). In the 2XY subset, except for the AA, TT, CC, and GG categories—which had lower S_2_ values—the 12 remaining dinucleotide categories formed an oblique line, indicating relatively small differences among them (Figure 9E,F). Taken together, the 2XY subset showed the highest S_2_ values, reflecting the greatest deviation from the overall 8-mer sequence; the 0XY subset had the lowest S_2_ values, indicating the least deviation; and the 1XY subset displayed intermediate values between the two.

### 2.8. Comparative Analysis of CG-Classified 8-mers Reveals Evolutionary Trends

**Figure 10:**
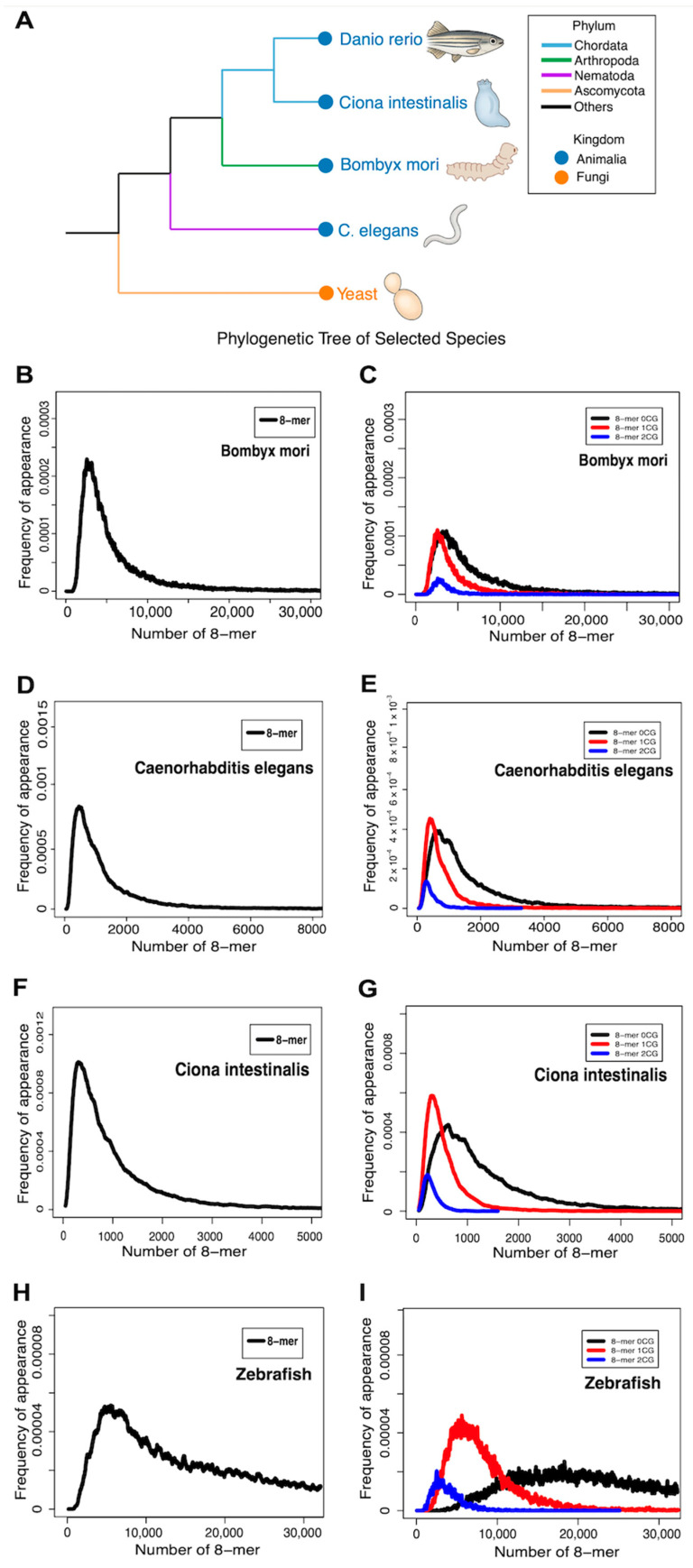
The 8-mer distributions in *Bombyx mori*, *Caenorhabditis elegans*, *Ciona intestinalis*, and zebrafish. (**A**) Phylogenetic tree of the species studied in this paper. (**B**) Overall 8-mer distribution in *Bombyx mori*. (**C**) Distribution of 8-mers containing 0, 1, or 2 CG dinucleotides in *Bombyx mori*. (**D**) Overall 8-mer distribution in *Caenorhabditis elegans*. (**E**) Distribution of 8-mers containing 0, 1, or 2 CG dinucleotides in *Caenorhabditis elegans*. (**F**) Overall 8-mer distribution in *Ciona intestinalis*. (**G**) Distribution of 8-mers containing 0, 1, or 2 CG dinucleotides in *Ciona intestinalis*. (**H**) Overall 8-mer distribution in zebrafish. (**I**) Distribution of 8-mers containing 0, 1, or 2 CG dinucleotides in zebrafish.

To explore the link between CG dinucleotide-classified 8-mers and species evolution, we compared *Saccharomyces cerevisiae* with *Bombyx mori*, *Ciona intestinalis*, *Danio rerio*, and *Caenorhabditis elegans*. Their phylogenetic tree is shown in Figure 10A. Unlike the trimodal distribution in human chromosome 1, all five species exhibited unimodal 8-mer distributions, with the 2CG and 1CG subsets shifted leftward relative to 0CG. The 1CG subset had a higher peak FA than both 0CG and 2CG, consistent with patterns in yeast and humans (Figure 10B–I). Peak distances between 0CG, 1CG, and 2CG subsets were calculated (Equations (9) and (10)), and significance was assessed by permutation test (Equations (11) and (12); Table 4 and Figure S1). The significance order for 0CG vs. 1CG and 0CG vs. 2CG was zebrafish > *B. mori* > *C. intestinalis* > *C. elegans* > yeast, suggesting that these distances may reflect evolutionary divergence. These findings suggest that the CG-classified 8-mer distribution patterns not only vary across species but also provide informative markers of phylogenetic divergence in species exhibiting unimodal 8-mer spectra.

## 3. Discussion

The non-random usage of k-mers and their biological functions have attracted increasing attention in genome research. Chor et al. analyzed k-mer distributions across 89 non-mammalian species and found that most display a Poisson-like unimodal pattern, whereas non-mammalian tetrapods tend to exhibit multimodal distributions—a trend that becomes predominant in mammals regardless of chromosome or genome scale. This shift has been associated with moderate GC content (35–45%) and low CpG dinucleotide ratios (<0.4) [24]. Previous studies further revealed a consistent trimodal distribution of k-mers (k > 6) in human intergenic regions, one that is particularly stable at k = 8. While the whole genome and non-coding regions exhibit three distinct peaks for k = 6 and 8, coding regions typically follow a unimodal 8-mer distribution [32]. These findings highlight the importance of investigating 8-mers containing CG dinucleotides, which may play key roles in shaping the compositional and functional landscape of the genome.

In this study, we investigated the distribution of 8-mers on human chromosome 1. The 0CG subset overlapped with the center of a randomized 8-mer distribution, whereas the 1CG and 2CG subsets were significantly underrepresented and skewed, suggesting non-random usage possibly driven by functional constraints. By analyzing the distribution of CG-containing 8-mers after removing CpG islands, we observed a marked change, particularly in 2CG 8-mers. This finding suggests that 8-mers with zero CpG dinucleotides (0CG) may result from random evolutionary processes, while 2CG 8-mers likely represent core motifs contributing to CpG island formation. Previous studies have shown that transcription factor binding sites and protein-binding sites in non-coding regions exhibit sequence preferences [42]. However, the number of such sites is insufficient to explain the substantial deviations observed in 1CG and 2CG 8-mers. A nucleosome unit, consisting of 146–170 bp of core DNA wrapped around a histone octamer and 6–80 bp of linker DNA, spans the majority of chromosomal DNA. The interaction between DNA and histones is influenced by sequence composition, indicating that base composition plays a critical role in nucleosome formation [43,44,45]. Additionally, GC-rich sequences have been implicated in nucleosome positioning, species evolution, and gene regulation [46,47,48,49,50,51,52]. Given the larger number of 1CG 8-mers, we propose that a subset of these motifs may be associated with histone interaction sites, potentially serving as the core elements of nucleosome-binding motifs. Notably, the trimodal distribution of 8-mer frequencies observed in our analysis may be partially attributed to the general under-representation of CG dinucleotides in vertebrate genomes. This phenomenon is largely due to the high mutation rate of methylated cytosine to thymine. Consequently, the low-frequency 1CG and 2CG peaks observed in human DNA are likely the result of this mutational bias [53].

Li et al. reported that only 8-mers containing CG or TA dinucleotides are directly associated with genome sequence evolution, and that the relative frequencies of internal m-mer usage are better suited to characterize this process [54,55]. To explore this further, we analyzed the m-mer usage separation of dinucleotide-containing 8-mers from a sequence structure perspective. We used NSRE(P‖Q), an information-theoretic measure of spatial state divergence [56], along with distance difference (S_1_) and angle deviation (S_2_), to quantitatively assess m-mer usage separation. Among the XY subsets, the 2XY group showed the highest separation, followed by 1XY and then 0XY. Within the 0XY group, 0CG exhibited the lowest separation, while 0AA and 0AT showed the highest. In the 1XY group, 1CG displayed the greatest separation, a pattern mirrored in the 2XY group, where 2CG had the highest separation. These results suggest that the evolutionary divergence of 0CG motifs may result mainly from random variation rather than m-mer usage preferences. In contrast, the divergence of 2CG motifs appears driven by biased m-mer usage or compositional skew. The 1CG motif represents an intermediate case, where divergence likely reflects a combination of low motif abundance and stochastic m-mer distribution rather than strong usage preference alone.

Through the comparative analysis of species exhibiting unimodal distributions, we found that the order of peak distance significance between 0CG vs. 1CG and 0CG vs. 2CG was as follows: *Danio rerio* > *Bombyx mori* > *Ciona intestinalis* > *C. elegans* > *Saccharomyces cerevisiae*. Yeast (*Saccharomyces cerevisiae*), a unicellular organism lacking tissue differentiation and relying on simple metabolic pathways such as fermentation, represents the lowest evolutionary tier among the species analyzed [57]. *C. elegans*, an invertebrate, is a multicellular organism with a fixed number of somatic cells (959 in adults) and possesses a simple yet functionally distinct nervous system [58]. Sea squirt (*Ciona intestinalis*), though equipped with a notochord and neural tube during its larval stage, undergoes regression into a sessile, filter-feeding adult form, resulting in a structurally simplified body plan [59]. This developmental reduction may partly account for why the peak distance significance between 0CG vs. 1CG and 0CG vs. 2CG in sea squirt is lower than that observed in silkworm. Silkworm (*Bombyx mori*), an invertebrate with specialized organs (e.g., compound eyes, silk glands), undergoes complete metamorphosis and displays greater anatomical complexity than simpler invertebrates, although it remains less advanced than vertebrates [60]. In contrast, zebrafish (*Danio rerio*), a vertebrate, occupies the highest evolutionary position in this group, characterized by well-developed organ systems, including a closed circulatory system, a complex central nervous system, and a functional adaptive immune system [61,62]. Zebrafish also exhibit complex behaviors, including social interactions [63]. Therefore, the hierarchical significance of the observed peak distances reflects the increasing structural and functional complexity among these species, suggesting that the evolutionary separation of 1CG and 2CG distributions may serve as an indirect indicator of evolutionary advancement in species exhibiting unimodal distributions.

Although some of our views have been supported by experiments and partially corroborated by the literature, certain aspects may still be difficult to accept. In our future work, we plan to address the following: (1) In this study, we focused on analyzing 8-mer sequences. We believe that conducting in-depth analyses of DNA sequences starting from k-mers (k ≥ 9) will provide further insights into the evolution and function of DNA sequences. (2) We calculated the degree of deviation in various subsets by comparing the usage differences of 3-mers and 4-mers between the subsets and the overall 8-mer dataset. The choice of 5-mers should be better explained in contrast to other options. For instance, Frontali et al. found that the choice of k = 5 could be equally relevant for preserving the “signal” properties of the k-mer distribution. In *Caenorhabditis elegans*, their recursive analysis revealed long-range correlations in the usage of oligonucleotides between intronic and intergenic regions [11,64]. (3) The NSRE is still a preliminary attempt and does not yet fully account for the differences caused by the high occurrence of m-mers with a relative frequency of 0. In the next phase, we will incorporate these factors to refine this parameter. (4) The separation of k-mer usage as a reflection of evolutionary relationships among biological genomes is a novel and significant topic. While this study compared only five representative unimodal species spanning fungi, invertebrates, and vertebrates, future research should include a broader range of organisms to further generalize the findings. (5) The hypothesis that 8-mers containing one CG dinucleotide may serve as nucleosome-binding motifs, and that those containing two CGs may function as core motifs involved in CpG island formation, remains preliminary. Notably, organisms such as *Saccharomyces cerevisiae* and *Caenorhabditis elegans* lack canonical CpG islands, suggesting that the functions of 2CG 8-mers in these species require further investigation [65]. In future work, we will systematically analyze the biological roles of both high-frequency 8-mers and those that occur less frequently. Addressing these open questions constitutes a key objective of our ongoing k-mer research.

In summary, this study systematically characterized the distribution patterns of 8-mer sequences containing CG dinucleotides in five unimodal species, revealing their non-random usage within these genomes. Integrating positional, structural, and evolutionary analyses, we identified distinct preferences for 1CG and 2CG motifs, which appear to correlate with organismal complexity within this limited set. These findings suggest that CG-containing k-mers may carry underlying regulatory or structural signals shaped by evolution. Our work provides new insights into the sequence-level features of genomic organization and offers a basis for further investigation into the biological functions of short DNA motifs.

## 4. Materials and Methods

### 4.1. Acquisition and Assembly of Whole Genome Sequences

Genome data for Homo sapiens chromosome 1 (230,481,012 bp) and all 16 chromosomes of *Saccharomyces cerevisiae* (Chr I–XVI; concatenated length 12,312,773 bp) were obtained from the UCSC Genome Browser (http://genome.ucsc.edu/, accessed on 16 September 2025). Whole-genome sequences of *Danio rerio* (zebrafish), *Bombyx mori* (silkworm), *Ciona intestinalis* (sea squirt), and *Caenorhabditis elegans* were retrieved from the NCBI genome database (https://www.ncbi.nlm.nih.gov/genome, accessed on 16 September 2025). Except for the human genome, species selection was based on two criteria: (i) motif spectra, as these organisms exhibit the unimodal 8-mer spectral distributions reported previously [49]; and (ii) phylogenetic representation, ensuring evolutionary breadth by including fungi (*S. cerevisiae*), invertebrates from distinct phyla (*B. mori*, *Arthropoda*; *C. elegans*, *Nematoda*; *C. intestinalis*, *Chordata*), and vertebrates (*D. rerio*). This design facilitates comparative analyses across major evolutionary lineages while relying on high-quality, well-annotated genomic resources.

### 4.2. Definition of k-mer Frequency of Appearance (FA)

*N_i_* refers to the number of k-mer motifs in the *i*-th frequency group. The arrangement of k-mers consists of 4*^k^* types. The frequency of appearance (FA) of a k-mer, that is, the relative motif count, is defined as follows:

(1) $$FA=\frac{N_{i}}{4^{k}}$$

by plotting the number of k-mer occurrences on the x axis and the *FA* of k-mers on the y axis, the distribution of *FA* of k-mers with respect to frequency is obtained.

### 4.3. Definition of the Relative Frequency of m-mers Within 8-mers

Each 8-mer contains (8 − m + 1) m-mers. Let *L_j_* represent the total number of 8-mers in the *j*-th set. The relative frequency (*RF*) of each m-mer is calculated as follows:

(2) $$RF=\frac{4^{m}}{\left(8-m+1\right)}\frac{\sum_{i=1}^{L_{j}}N_{mi}H_{i}}{\sum_{i=1}^{L_{j}}H_{i}}$$

In the above formula, *H_i_* represents the occurrence frequency of the *i*-th 8-mer in the *j*-th set, and *N_mi_* denotes the occurrence frequency of a specific m-mer within the *i*-th 8-mer in set *j*. In this study, *m* is 3 and 4.

### 4.4. Definition of New Symmetric Relative Entropy

Our previous work applied the New Symmetric Relative Entropy (NSRE) method to analyze nucleosome sequences in *S. cerevisiae*, *S. pombe*, and *Drosophila*. The NSRE distributions successfully captured characteristic differences in nucleosome sequences among these species [56]. NSRE quantifies the divergence of m-mer usage from an informational perspective by measuring the separation of m-mer frequencies between subsets (0XY, 1XY, 2XY) and the overall 8-mer set. This metric reflects the deviation of subset-specific 8-mers from the overall 8-mer distribution. Prior to NSRE calculation, the relative frequencies of m-mers in both subsets and the overall 8-mer set require normalization. Specifically, for any m-mer with a relative frequency of zero in a subset, its frequency is substituted with half of the smallest non-zero frequency observed in that subset. The NSRE between distributions *P* and *Q*, denoted as NSRE(P‖Q), is mathematically defined as follows:

(3) $$NSRE(P\left\|Q)\right.=\sum_{i=1}^{L_{m}}\left(p_{i}\log_{2}\frac{2p_{i}}{p_{i}+q_{i}}+q_{i}\log_{2}\frac{2q_{i}}{p_{i}+q_{i}}\right)$$

In the above formula, *p_i_* represents the relative frequency of the *i*-th m-mer in the subsets 0XY, 1XY, and 2XY, whereas *q_i_* denotes the relative frequency of the *i*-th m-mer in the overall set of 8-mers. For an information set composed of *L_m_* symbols, *L_m_* = 64 for 3-mers and *L_m_* = 256 for 4-mers. Both *p_i_* and *q_i_* are frequency values bounded between 0 and 1. If the relative frequency of a specific m-mer in a subset matches its frequency in the overall 8-mer set, its contribution to the NSRE is zero.

### 4.5. Definitions of Distance Difference and Angular Deviation

Standard deviation is a very important statistical measure that represents the degree of deviation of a variable series from its mean [66]. It is an indicator used to measure the dispersion of a variable series and is denoted as S*_x_*. We extended the form of standard deviation by introducing the concepts of distance deviation S_1_ and angular deviation S_2_:

(4) $$S_{x}=\sqrt{\frac{{\sum \left(x_{i}-\bar{x}\right)}^{2}}{n-1}}$$

(5) $$S_{1}=\sqrt{\frac{\sum_{i=1}^{n}d_{i}^{2}}{n-1}}$$

(6) $$d_{i}=\sqrt{p_{i}^{2}+q_{i}^{2}}\sin\alpha_{i}$$

(7) $$S_{2}=\sqrt{\frac{\sum_{i=1}^{n}\alpha_{i}^{2}}{n-1}}$$

(8) $$\alpha_{i}=\frac{\pi }{4}-\arctan\left(\frac{p_{i}}{q_{i}}\right)$$

where *p_i_* represents the relative frequency of the *i*-th m-mer in the subsets 0XY, 1XY, and 2XY, while *q_i_* denotes the relative frequency of the *i*-th m-mer in the overall set of 8-mers. For an information set composed of *n* symbols, *n* = 64 for 3-mers and *n* = 256 for 4-mers. Both *p_i_* and *q_i_* are frequency values ranging between 0 and 1. Here, *d_i_* is the vertical distance of the *i*-th m-mer from the diagonal line, and *a_i_* is the angle formed between the line connecting the origin and the *i*-th m-mer and the diagonal line. The distance deviation (S_1_) describes the separation of m-mer usage based on the vertical distance of the m-mer from the diagonal line. The angle deviation (S_2_) describes the separation of m-mer usage based on the angle formed by the line connecting the m-mer to the origin and the diagonal line.

### 4.6. Definition of Peak Distance Difference

Let $f_{0}\left(x\right)$, $f_{1}\left(x\right)$, and $f_{2}\left(x\right)$ represent the three frequency curves corresponding to 0CG, 1CG, and 2CG subsets, respectively. After smoothing, the main peak positions of these curves are identified as follows:

(9) $$\;{\hat{x}}_{i}=\arg\underset{x}{\max}f_{i}\left(x\right),i\in \left\{0,1,2\right\}$$

The peak distance between two frequency distributions can be defined as

(10) $$\;D_{ij}=\left|{\hat{x}}_{i}-{\hat{x}}_{j}\right|$$

### 4.7. Definition of Nonparametric Permutation Test

To test whether there is a significant difference between the main peak positions of two 8-mer subsets (i.e., whether the observed peak difference $D_{ij}^{obs}$ is significant), we first randomly shuffle the data labels of $f_{i}\left(x\right)$ and $f_{j}\left(x\right)$. After regrouping, we recalculate the smoothed curves and identify the peaks, obtaining the peak difference $D_{ij}^{\left(b\right)}$ for each permutation. After performing *B* permutations, a “null distribution” of $D_{ij}^{\left(b\right)}$ is constructed:

(11) $$D_{ij}^{\mathrm{null}}=\left\{D_{ij}^{\left(1\right)},D_{ij}^{\left(2\right)},\ldots ,D_{ij}^{\left(B\right)}\right\}$$

The corresponding *p*-value of the permutation test was calculated using the following Formula [67]:

(12) $$p=\frac{1}{B}\overset{B}{\underset{b=1}{\sum }}1\left(D_{ij}^{\left(b\right)}\geq D_{ij}^{\mathrm{obs}}\right)$$

The core idea of this method is to shuffle the labels of the pooled data, then recalculate the main peak difference, and finally repeat the process multiple times to generate a distribution under the null hypothesis for statistical evaluation.

## 5. Conclusions

- Human chromosome 1 exhibits a trimodal distribution of 8-mers classified by CG dinucleotide content, where 0CG motifs align with random distributions, while 1CG and 2CG motifs show significant deviations, indicating possible functional constraints.
- The unimodal distribution of 8-mers may arise from the close clustering of the distribution centers of 0CG, 1CG, and 2CG motifs.
- Comparative studies across species show that differences in CG-classified 8-mer distributions correlate with evolutionary complexity.

## Figures and Tables

**Table 1:** Genomic features of model organisms in 8-mer evolutionary research.

Species	Version	Sequence Length	Number of Chromosomes	Source
*Homo sapiens*	GCA_000001405.15 (GRCh38)	230,481,012 bp	1	UCSC
*Saccharomyces cerevisiae* (Yeast)	GCA_000146055.2 (SacCer3)	12,312,773 bp	16	UCSC
*Caenorhabditis elegans*	GCF_000002985.6 (WBcel235)	100,272,607 bp	6	NCBI
*Danio rerio* (Zebrafish)	GCF_000002035.6 (GRCz11)	1,345,101,833 bp	25	NCBI
*Ciona intestinalis* (Sea squirt)	GCF_000224145.3 (KH)	78,296,155 bp	14	NCBI
*Bombyx mori* (Silkworm)	GCF_030269925.1 (ASM3026992v2)	461,688,958 bp	29	NCBI

Note: The number of chromosomes used in this study excludes sex chromosomes and mitochondrial chromosomes.

**Table 2:** The relative frequencies of 3-mers in all 8-mer.

3-mer	RF	3-mer	RF
GCG	0.358	TAC	0.908
CGC	0.36	GTA	0.91
CGG	0.372	TGG	0.948
CCG	0.374	CCA	0.957
GGG	0.428	ACT	0.964
CCC	0.436	AGT	0.97
GGC	0.502	TGT	1.092
GCC	0.503	ACA	1.1
CGT	0.559	GAT	1.121
ACG	0.56	ATC	1.128
TCG	0.582	GTT	1.148
CGA	0.583	AAC	1.154
GTC	0.618	ATG	1.168
GAC	0.622	CAT	1.174
GTG	0.68	TCT	1.286
CAC	0.684	TGA	1.288
CTC	0.714	TCA	1.29
GAG	0.715	AGA	1.295
CCT	0.728	CTT	1.375
AGG	0.731	AAG	1.386
GCT	0.731	TTA	1.43
AGC	0.734	TAA	1.433
GGT	0.736	TTG	1.47
ACC	0.745	CAA	1.48
TGC	0.791	TTC	1.508
GCA	0.795	GAA	1.52
CTG	0.801	TAT	1.59
CAG	0.804	ATA	1.596
TCC	0.811	ATT	1.885
GGA	0.812	AAT	1.893
CTA	0.819	TTT	2.503
TAG	0.824	AAA	2.519

Note: RF stands for relative frequency.

**Table 3:** The relative frequencies of the rare and optimal 4-mer in all 8-mer.

4-mer Rare	RF	4-mer Optimal	RF
CGCG	0.187	CAAA	2.05
CGGG	0.237	TTCT	2.055
CCCG	0.245	TCTT	2.063
GCGC	0.288	AGAA	2.077
CCGG	0.298	AAGA	2.086
GGGG	0.308	TTTC	2.197
CCCC	0.318	GAAA	2.21
GCCG	0.324	TATT	2.239
CGGC	0.325	ATAT	2.258
CCGC	0.329	AATA	2.262
GCGG	0.331	AATT	2.361
GGCG	0.348	ATTT	2.691
GGGC	0.351	AAAT	2.708
CGCC	0.353	TTTT	3.756
GCCC	0.356	AAAA	3.786

Note: RF stands for relative frequency.

**Table 4:** Observed peak distances and permutation *p*-values for CG-based 8-mer comparisons across species.

Species	Observed Distance (0CG vs. 1CG)	Observed Distance (0CG vs. 2CG)	Observed Distance (1CG vs. 2CG)	Permutation *p*-value (0CG vs. 1CG)	Permutation *p*-Value (0CG vs. 2CG)	Permutation *p*-Value (1CG vs. 2CG)
*Yeast*	71	95	24	0.3098	0	0.0015
*Bombyx mori*	1074	1037	37	0.0575	0	0.4764
*Caenorhabditis elegans*	211	318	107	0.4357	0	0
*Ciona intestinalis*	317	403	86	0.0259	0	0
*Zebrafish*	12,675	15,784	3109	0	0	0

Note: A value of 0 for the permutation *p*-value indicates *p* ≤ 0.01.

## Data Availability

The whole-genome data used in this study were obtained from the UCSC (http://genome.ucsc.edu/, accessed on 16 September 2025) and NCBI (https://www.ncbi.nlm.nih.gov/, accessed on 16 September 2025) databases. Detailed version information for each dataset can be found in Table 1. The data supporting the figures and the code used in this study are available from the corresponding author upon reasonable request.

## Supplementary Materials

The following supporting information can be downloaded at https://www.mdpi.com/article/10.3390/ijms26199477/s1.

## Abbreviations

FA: Frequency of appearance; RF: Relative frequency; NSRE: New Symmetric Relative Entropy; m-mer: Short k-mer within 8-mer; S_1_: Distance difference; S_2_: Angular deviation; 0XY: 8-mers without XY dinucleotide; 1XY: 8-mers including 1 XY dinucleotide; 2XY: 8-mers including 2 or more than 2 XY dinucleotides.
