# Supplementary material for: CG-Based Stratification of 8-mers Highlights Functional Roles and Phylogenetic Divergence Markers
Source: Int J Mol Sci. 2025 Sep 27;26(19):9477. doi: 10.3390/ijms26199477 (PMC12525110; doi:10.3390/ijms26199477)
Supplement: Supplementary file 1 [file ijms-26-09477-s001.zip › ijms-3694973-supplementary-Figure S1.pdf]

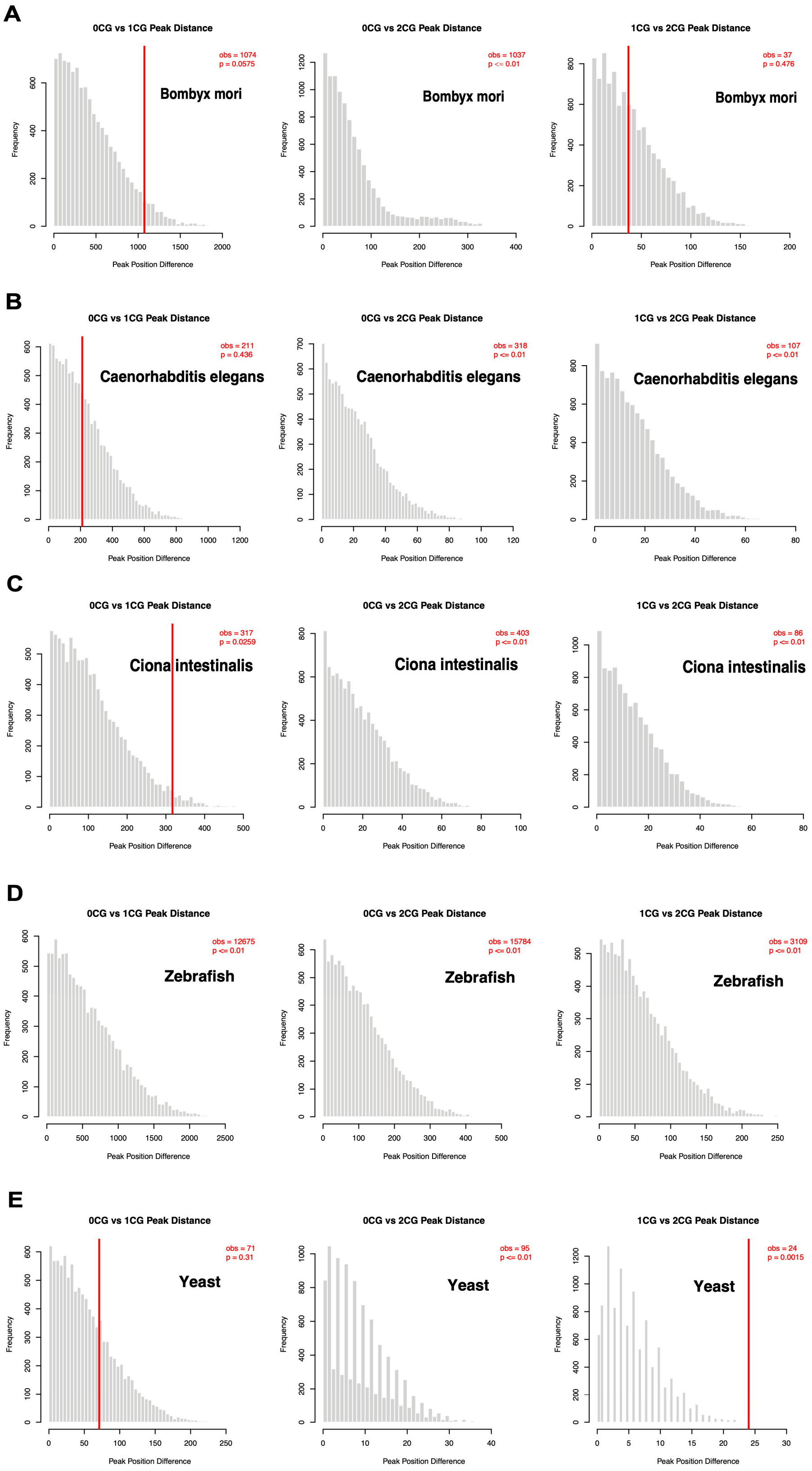


**Figure S1. Peak distance differences in the distributions of 0CG, 1CG, and 2CG 8-mers across the compared species.** (A) Peak distance differences in the distributions of 0CG, 1CG, and 2CG 8-mers in *Bombyx mori.* (B) Peak distance differences in the distributions of 0CG, 1CG, and 2CG 8-mers in Caenorhabditis elegans. (C) Peak distance differences in the distributions of 0CG, 1CG, and 2CG 8-mers in Ciona intestinalis. (D) Peak distance differences in the distributions of 0CG,

1CG, and 2CG 8-mers in Zebrafish. (E) Peak distance differences in the distributions of 0CG, 1CG, and 2CG 8-mers in Yeast.
