# Supplementary material for: CG-Based Stratification of 8-mers Highlights Functional Roles and Phylogenetic Divergence Markers
Source: Int J Mol Sci. 2025 Sep 27;26(19):9477. doi: 10.3390/ijms26199477 (PMC12525110; doi:10.3390/ijms26199477)
Supplement: Supplementary file 1 [file ijms-26-09477-s001.zip › ijms-3694973-supplementary-Table_S1.pdf]

Table S1. Statistical summary of rare and frequent 8-mers in the yeast genome.

INFREQUENTLY

CGCGTGAC(4)	GACCCGCG(4)	CCCCGGGG(5)	ACGCCCCG(6)
GCGCCCCG(6)	TCCCCGGG(6)	TCGGGGGG(6)	CCCCCCGA(7)
CCGGGGGG(7)	CGCGGTCC(7)	CGGGGGGC(7)	GCCCGGGG(7)
CCCGGGGG(8)	CGCCCGGG(8)	CGCGCCGA(8)	CGGCCCAC(8)
CGGGGCCG(8)	GACTCGCG(8)	ACGGGGCG(9)	AGCCCGGG(9)
CCCGCGTC(9)	CCCGGGGC(9)	CCCGGGTC(9)	CCGGGCGT(9)
CGCGCCCC(9)	CGCTCGCG(9)	CGCTCGGG(9)	CGGGACGC(9)
GCCCCGTC(9)	GCGCGCCG(9)	GGGCCGCG(9)	GGGTCGCG(9)
TCCCGCGC(9)	ACCCCGGG(10)	ACGGTCCG(10)	CCGACCGC(10)
CCGACCGG(10)	CCGCGTGC(10)	CCGGGCCG(10)	CCGGGGGT(10)
CGCCCCGA(10)	CGCCCCGG(10)	CGCGCTCG(10)	CGGACCGT(10)
CGGCCGCG(10)	CGGCGCGT(10)	CGGGGGAC(10)	GCCCCGGG(10)
GCCGGGGG(10)	GCGCTCCG(10)	GGGCCGGG(10)	GGGGCGCG(10)

FREQUENTLY

TTTTATTT(1787)	AAATAAAA(1802)	TTTTTATT(1804)	TTTATTTT(1807)
TTATTTTT(1808)	AAAATTTT(1826)	AATAAAAA(1832)	TATTTTTT(1841)
TTCTTCTT(1864)	AAAAATTT(1868)	AAAATAAA(1868)	AAAAAAGA(1874)
AAAAATAA(1886)	TAAAAAAA(1908)	TTTTTTCT(1935)	TTTTTTTA(1936)
AAAAAATA(1941)	AAGAAGAA(1942)	AATTTTTT(1945)	AATAATAA(1946)
AGAAAAAA(1953)	AAAAAATT(1978)	CTTTTTTT(2166)	AAAAAAAG(2203)
AAAAAGAA(2243)	TTCTTTTT(2256)	TTTTTCTT(2404)	AAGAAAAA(2462)
TTTTTTTC(2512)	AAAAGAAA(2515)	AAAGAAAA(2533)	TTTTCTTT(2548)
TTTCTTTT(2566)	GAAAAAAA(2571)	AAAAAAAT(2781)	ATTTTTTT(2826)
TATATATA(3892)	ATATATAT(4002)	TTTTTTTT(8529)	AAAAAAAA(8566)

Note: The number in parentheses indicates the number of occurrences of this 8-mer in the distribution.
